# Long term results after fractionated stereotactic radiotherapy (FSRT) in patients with craniopharyngioma: maximal tumor control with minimal side effects

**DOI:** 10.1186/1748-717X-9-203

**Published:** 2014-09-16

**Authors:** Semi B Harrabi, Sebastian Adeberg, Thomas Welzel, Stefan Rieken, Daniel Habermehl, Jürgen Debus, Stephanie E Combs

**Affiliations:** Department of Radiation Oncology, University Hospital of Heidelberg INF 400, 69120 Heidelberg, Germany; DKFZ Clinical Cooperation Unit Radiation Oncology, German Cancer Research Center, Heidelberg, Germany

**Keywords:** Craniopharyngioma, FSRT, Long-term results, Side effects, Radiological outcome

## Abstract

**Purpose:**

There are already numerous reports about high local control rates in patients with craniopharyngioma but there are only few studies with follow up times of more than 10 years. This study is an analysis of long term control, tumor response and side effects after fractionated stereotactic radiotherapy (FSRT) for patients with craniopharyngioma.

**Patients and methods:**

55 patients who were treated with FSRT for craniopharyngioma were analyzed. Median age was 37 years (range 6–70 years), among them eight children < 18 years. Radiotherapy (RT) was indicated for progressive disease after neurosurgical resection or postoperatively after repeated resection or partial resection. A median dose of 52.2 Gy (50 – 57.6 Gy) was applied with typical dose per fraction of 1.8 Gy five times per week. The regular follow up examinations comprised in addition to contrast enhanced MRI scans thorough physical examinations and clinical evaluation.

**Results:**

During median follow up of 128 months (2 – 276 months) local control rate was 95.3% after 5 years, 92.1% after 10 years and 88.1% after 20 years. Overall survival after 10 years was 83.3% and after 20 years 67.8% whereby none of the deaths were directly attributed to craniopharyngioma. Overall treatment was tolerated well with almost no severe acute or chronic side effects. One patient developed complete anosmia, another one’s initially impaired vision deteriorated further. In 83.6% of the cases with radiological follow up a regression of irradiated tumor residues was monitored, in 7 cases complete response was achieved. 44 patients presented themselves initially with endocrinologic dysfunction none of them showed signs of further deterioration during follow up. No secondary malignancies were observed.

**Conclusion:**

Long term results for patients with craniopharyngioma after stereotactic radiotherapy are with respect to low treatment related side effects as well as to local control and overall survival excellent.

## Background

Craniopharyngioma are rare epithelial solid or mixed solid-cystic tumors which arise from remnants of the Rathke pouch mostly in the sellar or suprasellar region. Despite its benign histological appearance as WHO grade I tumor they often recur and lead to significant clinical impairment by compression of structures in close vicinity such as optic pathways, hypothalamus or pituitary stalk. Craniopharyngioma account for 1–3% of all intracranial neoplasm [1] and most commonly present in children between 5 and 14 years of age or again in adults between 50 and 75 years [2, 3]. Both neurosurgical and radiotherapeutical approaches are well established in the treatment of craniopharyngioma however the optimal treatment strategies are still discussed controversially [4, 5]. Surgery is usually considered the treatment of choice, especially in children. The extent of microsurgical resection is most important for long-term local control [6] however gross total resection often cannot be achieved without leading to severe hypothalamic-pituitary dysfunction or visual impairment [7]. Furthermore limited resection can be necessary to relieve acute symptoms of intracranial pressure. In the past, radiotherapy (RT) was applied mostly adjuvant after surgery or in case of recurrence however there are plenty studies providing data that RT alone is as effective in respect to local control [8–11]. Since life expectancy is long local control assumed minimizing long term treatment related side effects is of highest priority. Using modern techniques of RT such as fractionated stereotactic radiotherapy (FSRT), stereotactic radiosurgery (SRS) or treatment with charged particles enables the delivery of high doses necessary for tumor control while not exceeding tolerance doses of nearby risk organs such as the brainstem or optic chiasm and thereby preventing further endocrinologic or visual deficits; moreover, reduction of low to medium doses to adjacent normal tissue can be achieved. In previous studies we reported of safety and effectiveness of FSRT in patients with craniopharyngioma [12]. The current study is a retrospective analysis of updated long-term clinical outcome and treatment-related side effects with particular regard to tumor response.

## Methods and material

### Patient characteristics

We evaluated 55 patients (28 female, 27 male, including 8 children < 18 years) who have been treated between 1989 and 2012 with FSRT for craniopharyngioma at our institution. Median age was 37 years (range 6 – 70). In all patients diagnosis was histologically confirmed. The majority of patients (70.9%, 39 of 55 patients) underwent RT with tumor progression/recurrence after surgery rather than adjuvantly after partial resection (29.1%, 16 of 55 patients).

### Treatment planning

All patients were immobilized by utilizing an individually manufactured mask system. In most cases an individually adapted scotch cast mask allowing a repositioning accuracy of 1 to 2 mm was used [13]. From 1993 on all patients received both CT and MRI with slice thickness of 3 mm in mask fixation for treatment planning as described previously [12]. Before then, only CT scans with stereotactic localization system were performed. After stereotactic guided image fusion (gross) target volume (GTV) was defined on each slice by outlining contrast enhanced T1-weighted solid lesions as well as all cystic components and their wall apparent in T2-weighted MRI scans. We defined the clinical target volume (CTV) for craniopharyngioma to be identical with the GTV. For safety reasons in regard to positioning accuracy a 2 mm margin was added for planning target volume (PTV). Treatment planning was performed by using the Stryker-LeibingerSystem STP, the Virtuos-Software or the Precisis-Software.

### Radiotherapy

Radiotherapy was conducted at our institution using linear accelerators with 6 or 15 megaelectron volts designed for stereotactic RT. Details on treatment planning and dose calculation were reported previously at our institution [14]. A median dose of 52.2 Gy (range 50 – 57.6 Gy) was applied in conventional fractionation with a median single dose of 1.8 Gy (range 1.8 – 2 Gy) 5 times per week using 3–5 individually collimated, non-coplanar, isocentric fields. The median PTV treated was 31.2 ml (range 6.2 to 138.9 ml). To account for possible changes of the target volume MRI scans were performed once during treatment in asymptomatic patients. In case of new clinical symptoms CT or MR imaging was performed more frequently.

### Radiological assessment

Radiological outcome was classified in five grades by assessing maximal axial dimension in two orthogonal directions: complete response (CR) was defined as absence of detectable tumor residuum on MRI scans. Tumor diminution of > 50% was assessed as partial response (PR) and in case of reduction of tumor formation between 25 and 50% as objective response. Change of dimensions of less than 25% without signs of progression was categorized as stable disease. Additionally, similar to treatment planning all solid and cystic components visible on follow up MRI were taken into consideration and outlined on each slice separately to gain volumetric information and put them in relation to the pretherapeutic GTV.

### Follow-up

All follow-up appointments consisted of complete physical examinations and clinical evaluation of endocrinologic, ophthalmologic and neurologic status in addition to neuroradiologic imaging. First visit was scheduled six weeks after completing RT, then patients were seen every quarter in the first year and every six months in the following years. Five years after RT intervals were prolonged to once a year. In case of follow up was continued at another facility our data was completed by consulting the attending physicians or contacting the patients themselves. Acute or chronic treatment related side effects were classified under the terms of common terminology criteria of adverse events (CTCAE, v4.043, June 14, 2010, available at http://evs.nci.nih.gov/ftp1/CTCAE/About.html).

### Statistical analysis

Overall survival was calculated from the time of first diagnosis up to the last follow-up visit or death by any cause. Local control rate was calculated from the onset of radiotherapy using the Kaplan-Meier method. (Sigma Plot, Systat Software GmbH).

## Results

### Radiotherapy

Radiotherapy was completed in all 55 patients as intended. 39 patients underwent treatment because of recurrent or progressive craniopharyngioma after initial resection. The mean time between surgery and begin of radiotherapy was 40.7 months (range 6 – 256 months). Due to hydrocephalus after cyst enlargement treatment had to be interrupted in one patient after only two fractions. With a newly implanted ventriculo-peritoneal shunt in place and updated postoperative cerebral imaging treatment was replanned and could be continued without further complications. Apart from this case there were no other significant adverse events or incidents exceeding grade I according to CTCAE.

### Follow up

Median follow up time was 128 months (range 2 – 276 months). During this period two female patients (29 years, 51 years) presented with signs of shunt insufficiency three and 49 months after RT necessitating neurosurgical intervention. In both patients no progress of the craniopharyngioma could be seen by means of MRI. Due to perioperative complications one patient died after only two months of follow up and the other’s general condition deteriorated that no further MRI scans were performed hence the patient was censored after 49 months dying 88 months after RT. A third 25 year old female patient developed 21 months after RT focal neurological deficits, nausea and emesis as well due to shunt insufficiency. Once the ventriculo-peritoneal shunt was replaced the symptoms disappeared and no further interventions were necessary. At her last follow-up she was in good general condition with stable disease.

After receiving FSRT improvement of visual impairment was notable in six patients and only one 56 year old female patient who developed mild decrease of visual acuity. Prior to RT 44 patients suffered from endocrinopathies. It is worth mentioning that all patients had intact hormone function at the time of initial diagnosis and developed endocrinological deficiencies only postoperatively.

**Figure 1:**
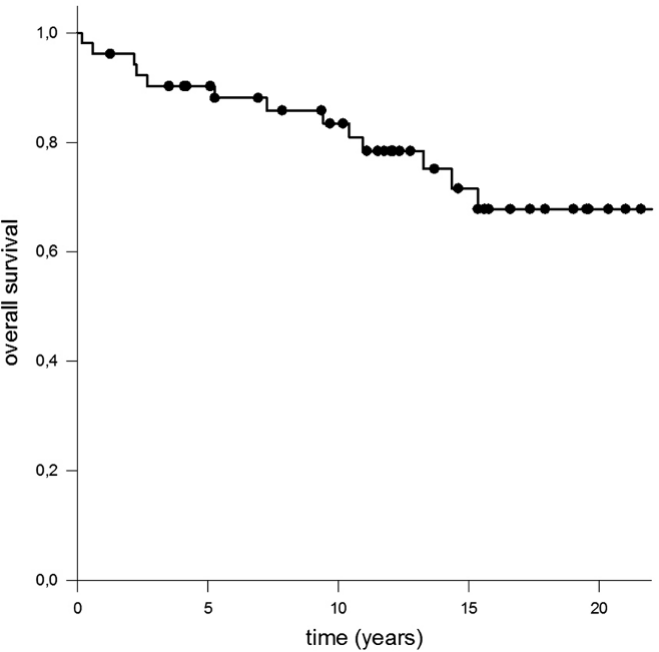
**Median overall survival was 90.3% after 5 years respectively 83.3% (10 years) and 67.8% (20 years).**

**Figure 2:**
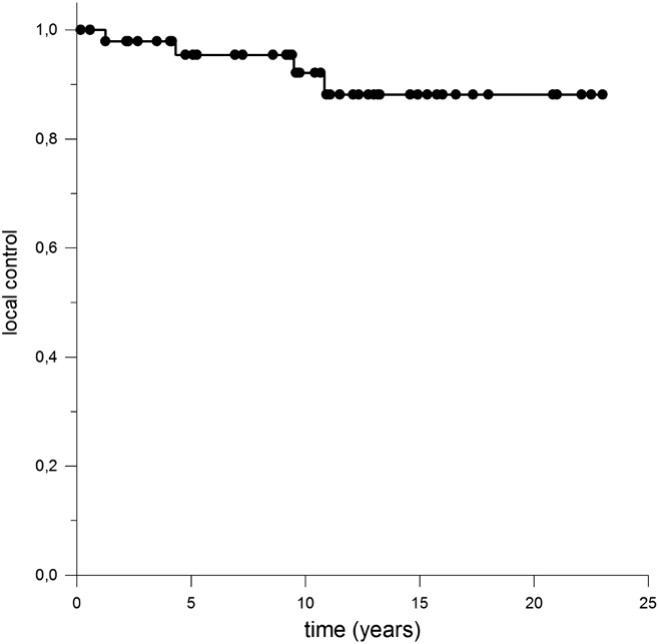
**Local control rate was 95.3% after 5 years, 92.1% after 10 years and 88.1% after 20 years.**

**Figure 3:**
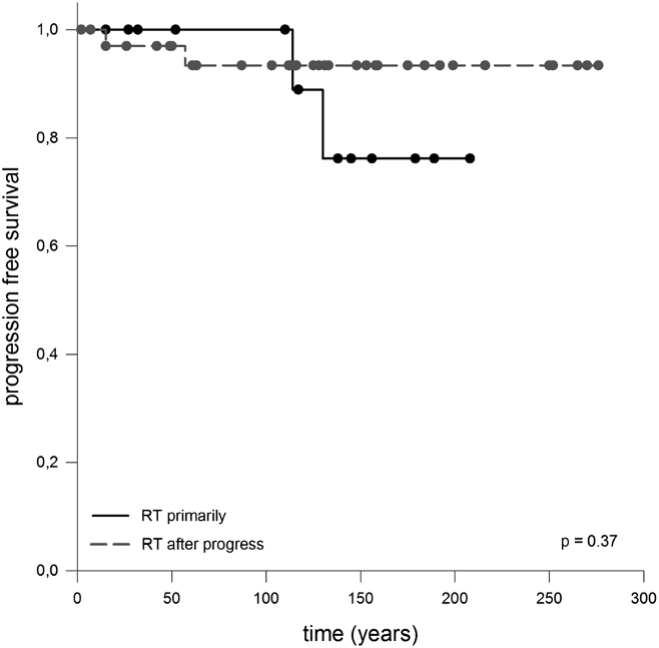
**No significant difference was observed for progression free survival whether patients were treated early in the time course or received RT for tumor recurrence, p-value = 0.37.**

One 50 year old male patient reported during follow up of progredient loss of sense of smell leading to complete anosmia 26 months after RT. Impairment of cognitive function did not occur however formal neuropsychological testing was not performed. Assessment of neurologic impairment was based on information provided by the patients and their home physicians e.g. whether change in social behavior appeared or special educational needs were required. Furthermore no severe treatment related long term side effects were observed, especially no case of radionecrosis or secondary malignancies.Median overall survival was 90.3% after 5 years respectively 83.3% (10 years) and 67.8% (20 years) whereby none of the deaths were directly attributed to craniopharyngioma (Figure 1). Local control rate was 95.3% after 5 years, 92.1% after 10 years and 88.1% after 20 years (Figure 2). There was no significant difference notable when overall survival was calculated in regard to adjuvant RT opposed to RT in case of progression, p-value = 0.37 (Figure 3).

### Radiological assessment

**Figure 4:**
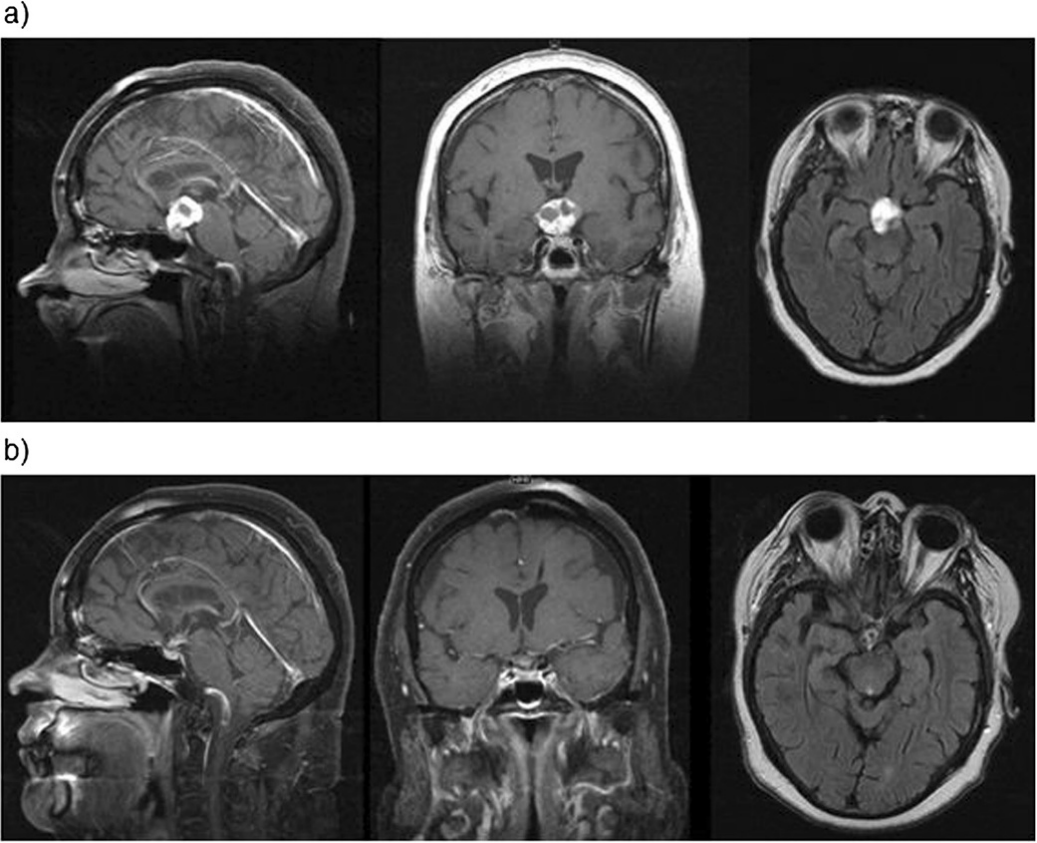
**Comparison of representative sagittal, coronar and transversal images from pre- and posttherapeutic MR scans of a 56 year-old female patient before (a) and six months after radiotherapy (b): a substantial decrease of the solid-cystic tumor can be detected leaving only small residues.**

After six months in 83.6% of the cases with radiological follow up a regression of irradiated tumor residues was monitored, in eight cases complete response was achieved (Figure 4, Table 1). Additionally, where available images from restaging MRI were uploaded to treatment planning system and GTV was again defined by outlining contrast enhanced T1-weighted solid lesions as well as all cystic components and their wall apparent in T2-weighted MRI scans allowing an assessment of pre- and post-therapeutic tumor volume. Treatment of craniopharyngioma with FSRT led to a median volume reduction of 60.8%.

**Table 1:** **Radiological outcome 6 months after radiotherapy (as defined in methods and material)**

Complete response	7 patients
Partial response	34 patients
Objective response	8 patients
Stable disease	5 patients
Progress	1 patient

## Discussion

Our study shows that long term outcome for patients with craniopharyngioma after being treated with FSRT is excellent as low treatment related side effects is concerned as well as local control rate and overall survival.

Optimal treatment strategy for craniopharyngioma has been subject to debate for a long time and is still discussed controversially [6, 12, 15, 16]. In the past, the recommended therapeutical approach consisted of attempted complete resection. However the tumor’s vicinity to vital structures such as the hypothalamus or the optic chiasm posed a major challenge for achieving this goal without inflicting severe morbidity [17, 18]. Even with advances of neurosurgical techniques such as implementation of microsurgery a primarily gross total resection is often associated with poor results with regard to endocrinological or neurocognitive outcome [7, 19]. In recent years less radical surgical approaches in combination with adjuvant treatment established [20]. Especially in cases of subtotal resection RT has been performed with similar results in terms of local control and overall survival [21, 22]. However each strategy has its distinct treatment related risk profile; neurosurgical resection is associated with acute complications whereas RT-induced side effect may occur only after many years.

**Figure 5:**
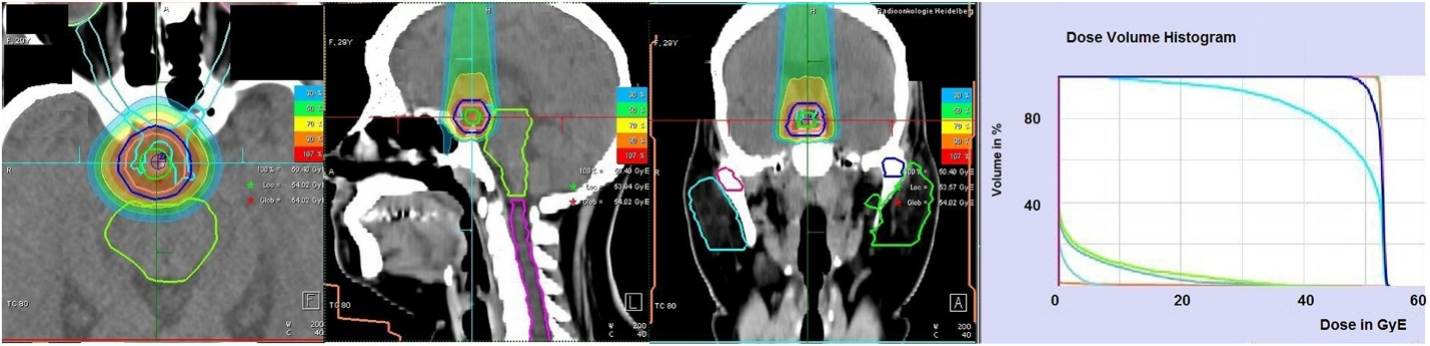
**Exemplary dose volume histogram of a FSRT plan with a proton beam.** Significant reduction of integral dose and thereby potentially reducing especially long-term side effects can be achieved.

There is controversial discussion about the optimal dose for craniopharyngioma. As it is a benign disease, one is reluctant to apply high doses. However a total dose of > 54 Gy is recommended for external radiation using conventional techniques with excessively rising recurrence rates for doses < 54 Gy [23]. Limitations are posed by the tolerance doses of vital organs in the vicinity. Therefore, commonly doses between 50.4 – 54 Gy are applied, depending on the volume of the lesion, the anatomical pattern, pretreatment factors as well as physician and center preference. Technical advances and modern techniques such as FSRT deliver means for applying high doses to a specific target while sparing the surrounding risk organs. In addition fractionation causes further radiobiological advantages concerning treatment-related adverse reactions [24]. Previous analysis of 40 patients at our institution resulted in excellent local control of 100% and overall survival of 89% after 10 years [12]. Similar findings were reported in other studies [25, 26]. Since life expectancy for patients with craniopharyngioma after receiving treatment is barely limited the reduction of long term treatment-related side effects is of highest relevance. Our long term follow-up of 128 months combined with low rates of acute and chronic side effects reinforces the role of FSRT in the management of craniopharyngioma. Considering the excellent long-term prognosis it can be argued if further improvement by proton therapy is possible. With protons, low doses are deposited within the entry channel of the beam, and high local dose deposition can be achieved within the Bragg peak. Due to these physical properties, reduction of integral dose is possible (Figure 5) potentially reducing especially long-term side effects, such as neurocognitive dysfunctioning, hormonal deficiency and secondary malignancies even more.

Yet the point in time of RT is still disputed. Should RT be reserved for tumor recurrence or rather be administered at first diagnosis not least because of excellent functional outcome? Regimen et al. reported of improved overall survival when treated primarily [27]. Moon et al. recently suggested comparable results in overall survival for adjuvant RT, early or late [28]. Similar findings were made by Pemberton et al. [29]. The data provided by our analysis provide the evidence that FSRT is a safe and effective treatment option for achieving long term control without inflicting severe functional or hormonal impairment. Whether patients were treated early in the time course or received RT for tumor recurrence did not seem to make a difference for local control, overall survival or neurocognitive function.

With FSRT a reduction of safety margins is possible, thereby leading to a smaller PTV and sparing of normal tissue [30]. The main rationale for target volume reduction is to decrease radiation induced long term complication such as secondary malignancies or impaired cognitive function. Particle therapy with protons offer several potential biological and physical advantages such as inverse depth dose profile leading to a steep dose gradient around the target volume. Thereby especially the low and medium dose regions could be minimized. Fitzek et al. presented an analysis of 15 patients treated with a combination of photon and proton irradiation for craniopharyngioma resulting in local control rates and overall survival comparable to other contemporary investigations [31]. However the risk of new cyst formation and therewith alteration of PTV has to be taken into consideration for proton therapy.

## Conclusion

FSRT leads to excellent results in patients with craniopharyngioma regarding local control, overall survival and preservation of organ function. Proton therapy may be able to further enhance the risk-benefit profile. Interdisciplinary treatment of these patients, especially in pediatric age, is strongly recommended to optimally decide on the sequence between wait-and-scan, surgery and/or radiation therapy.

### Consent

Written consent was obtained from the patient or the patient’s parents for the publication of this report and any accompanying images.
